# The role of HDAC2 in chromatin remodelling and response to chemotherapy in ovarian cancer

**DOI:** 10.18632/oncotarget.6618

**Published:** 2015-12-14

**Authors:** Rui Huang, Simon P Langdon, Matthew Tse, Peter Mullen, In Hwa Um, Dana Faratian, David J Harrison

**Affiliations:** ^1^ Division of Pathology, Institute of Genetics and Molecular Medicine, University of Edinburgh, Edinburgh EH4 2XU, UK; ^2^ School of Medicine, University of St Andrews, St Andrews, Fife, KY16 9TF, UK

**Keywords:** HDAC2, chromatin, platinum, ovarian cancer

## Abstract

Chromatin undergoes structural changes in response to extracellular and environmental signals. We observed changes in nuclear morphology in cancer tissue biopsied after chemotherapy and hypothesised that these DNA damage-induced changes are mediated by histone deacetylases (HDACs). Nuclear morphological changes in cell lines (PE01 and PE04 models) and a xenograft model (OV1002) were measured in response to platinum chemotherapy by image analysis of nuclear texture. HDAC2 expression increased in PEO1 cells treated with cisplatin at 24h, which was accompanied by increased expression of heterochromatin protein 1 (HP1). HDAC2 and HP1 expression were also increased after carboplatin treatment in the OV1002 carboplatin-sensitive xenograft model but not in the insensitive HOX424 model. Expression of DNA damage response pathways (pBRCA1, γH2AX, pATM, pATR) showed time-dependent changes after cisplatin treatment. HDAC2 knockdown by siRNA reduced HP1 expression, induced DNA double strand breaks (DSB) measured by γH2AX, and interfered with the activation of DNA damage response induced by cisplatin. Furthermore, HDAC2 depletion affected γH2AX foci formation, cell cycle distribution, and apoptosis triggered by cisplatin, and was additive to the inhibitory effect of cisplatin in cell lines. By inhibiting expression of HDAC2, reversible alterations in chromatin patterns during cisplatin treatment were observed. These results demonstrate quantifiable alterations in nuclear morphology after chemotherapy, and implicate HDAC2 in higher order chromatin changes and cellular DNA damage responses in ovarian cancer cells *in vitro* and *in vivo*.

## INTRODUCTION

Chromatin structure is dynamic, and changes occur in response to extracellular and environmental signals [1]. Histone tail acetylation is an important chromatin modification that alters DNA accessibility to regulating enzymes by transforming chromatin from a compact to relaxed structure that is permissive of gene expression [2, 3]. The balance between histone acetyltransferase (HAT) and histone deacetylase (HDAC) activities ultimately determines acetylation status [4]. Histone acetylation is involved with cellular differentiation, mitosis and meiosis, DNA transcriptional regulation, DNA damage, DNA replication, and circadian rhythms [5, 6, 7, 8].

Mammalian HDACs are grouped into four classes based on structural homology, enzymatic activity, and cellular localisation [9, 10, 11, 12]. Class I HDACs (HDAC 1, 2, 3, and 8) are mainly nuclear, and they interact with histones and other proteins [13, 14], while class II HDACs (HDAC 4, 5, 6, 7, 9, and 10) are tissue-specific and can be both nuclear and cytoplasmic [13]; the majority of HDAC inhibitors inhibit both class I and class II enzymes [15, 13]. Class III HDACs, namely sirtuins (SIRTs1 – 7; silent information regulators), are unresponsive to most HDAC inhibitors but require the cofactor NAD+. Finally, the class IV HDAC, HDAC11, is expressed in the nucleus and shares homology with class I and class II HDACs [15, 16].

HAT and HDAC activity can be altered by mutation, overexpression, or translocation, disrupting the acetylation-deacetylation balance and consequently contributing to cancer hallmarks; these epigenetic changes have been observed in leukaemia and prostate, breast, colorectal, and ovarian cancers [17, 13]. Acetylation changes are thought to participate in carcinogenesis by silencing tumor suppressor gene promoters, such as p21, [18, 19], activation of repressed genes, or abnormal recruitment of HATs or HDACs [13].

The heterochromatin protein 1 (HP1) protein family plays various roles in establishing and maintaining heterochromatin (tightly-packed DNA) structure, thereby repressing transcription [20]. HP1 overexpression can cause global gene repression and chromatin condensation [21, 22]. The three human HP1 isoforms, HP1α, HP1β, and HP1γ, share functions and localise to chromatin with incomplete overlap [23], differentially localising to centric heterochromatin, telomeres, and specific euchromatic sites [24]. Alterations in HP protein expression have been identified in some cancers including ovarian [25], breast [26], and colorectal cancer [27].

We have observed changes in nuclear structure in clinical samples of cancer tissue after treatment with chemotherapy and radiotherapy. Since structure dictates gene expression and, therefore, function, we sought to investigate this phenomenon to better understand therapeutic responses. We hypothesised that nuclear morphological changes in cancer in response to DNA damage are mediated by HDACs and are associated with changes in HP1 protein expression and/or nuclear distribution. Initial studies indicated changes in expression of HDAC2, therefore, we explored whether HDAC2 mediated response to injury and might act as a resistance factor to DNA-damaging therapy.

## RESULTS

### Nuclear structure changes after chemotherapy or radiotherapy in ovarian cancer cells

**Figure 1:**
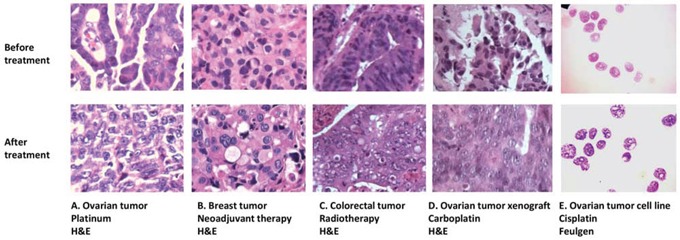
Nuclear morphology changes in different clinical and experimental settings **Similar nuclear texture changes occur in: A.** ovarian tumors after chemotherapy; **B.** breast tumors after neoadjuvant therapy; **C.** colorectal tumors after radiotherapy; **D.** ovarian tumor xenografts after carboplatin treatment; and E. the ovarian cancer cell line PEO1 after cisplatin treatment. A-D are formalin-fixed, paraffin embedded sections cut from tumor samples and stained with H&E. E shows PEO1 cell cytospins using the Feulgen nuclear stain.

Preliminary observations by light microscopy suggested that nuclear morphology was different in clinical tumors after treatment with chemotherapy and radiotherapy (Figure 1). To explore this phenomenon further, chromatin patterns were quantified by nuclear texture image analysis in an ovarian cancer cell line model. Texture features were regarded as positively or negatively associated with chromatin patterns (homogeneity, heterogeneity, and contrast) as previously described [28, 29]. Five parameters associated with texture were obtained: angular second moment (ASM), correlation, entropy, inverse different moment (IDM) and contrast. ASM is a strong measure of uniformity or smoothness associated with overall homogeneity of chromatin patterns. Correlation calculates the grey-level linear dependency of the image and correlates negatively with the heterogeneity of chromatin patterns. Entropy measures pattern disorder and is negatively correlated with homogeneity. Inverse different moment (IDM) measures the local variability and intensity of a region of interest (ROI) and is affected by image homogeneity, with non-homogeneous areas normally resulting in low IDM values; thus, it is described as the ‘contrast’ of chromatin patterns.

PE01 ovarian cancer cells [30] grown on coverslips were treated with cisplatin or ionising radiation and incubated for 0h, 6h, 12h and 24h. After 24 h treatment with 6 μM cisplatin or 6 Gy radiation, all five image texture parameters measured changed compared to untreated controls; observations were similar for both cisplatin and radiation. ASM, correlation, and IDM decreased after cisplatin/radiation treatment by 20%/23%, 25%/49%, and 15%/11%, respectively, while entropy and contrast increased by 6%/8% and 40%/120%. The heterogeneity and contrast of chromatin increased and the homogeneity decreased in cell nuclei after DNA damage-inducing treatment with cisplatin and radiation, consistent with the observations made by light microscopy.

### Measurement of nuclear texture changes in response to carboplatin in vivo

**Figure 2:**
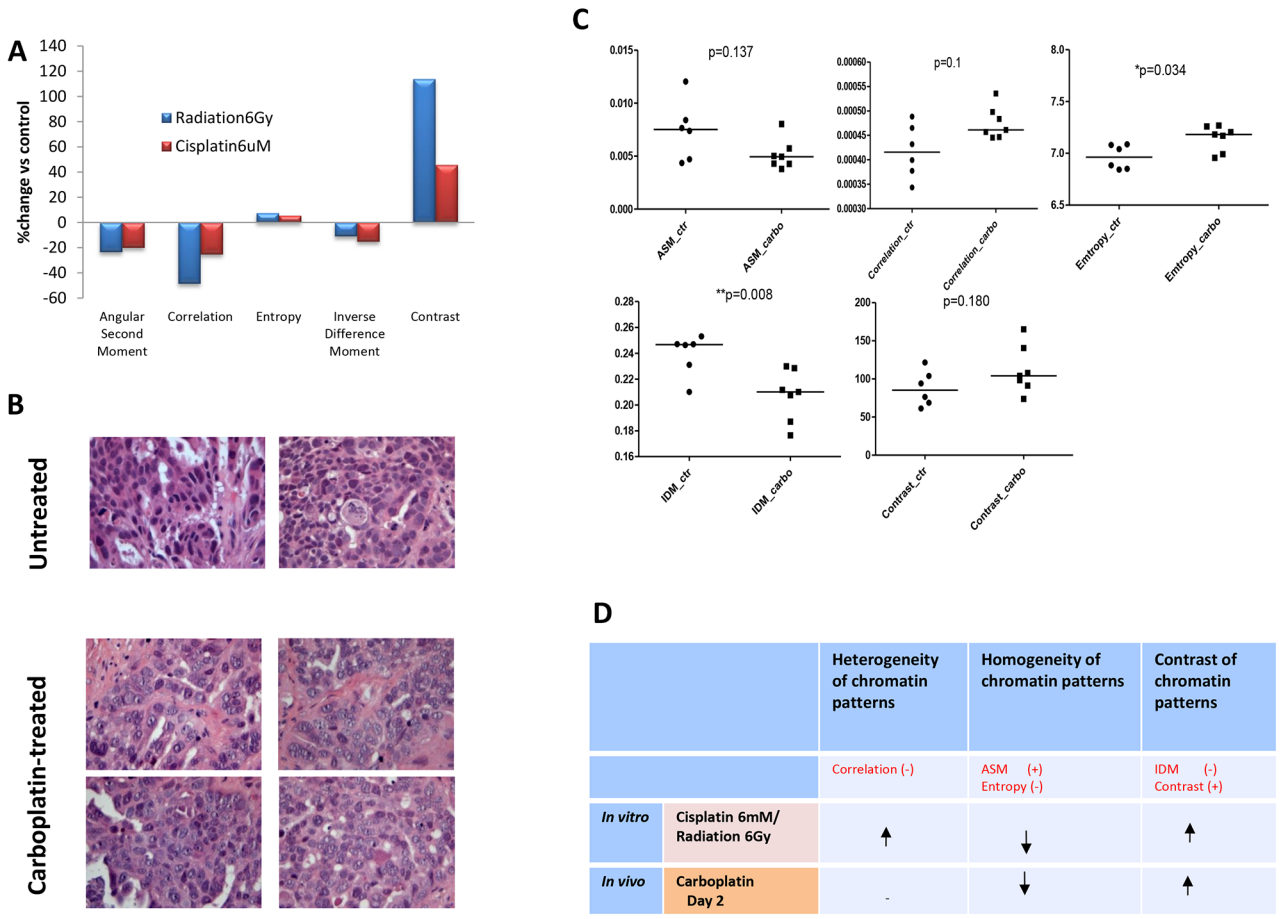
Alterations in nuclear texture in vitro and in vivo after treatment with platinum drugs or radiotherapy **A.** Changes in nuclear texture in PEO1 cells after irradiation or cisplatin treatment. PEO1 cells were grown on coverslips and treated with ionising radiation (6Gy) or cisplatin (6uM) for 24h, and nuclei were stained with DAPI for visualisation using a fluorescence microscope. At least 100 nuclei were included in each experiment. Nuclear texture was analysed by measuring five texture parameters (angular second moment, correlation, entropy, inverse different moment, and contrast) using Image J software. Data are presented as the average change (%) in the treated group for each parameter over the control group. **B.** Representative images from H&E-stained OV1002 ovarian tumor samples either untreated or after carboplatin (50 mg/kg) treatment in vivo. H&E stained images were acquired under 40x magnification. **C.** Nuclear texture parameter analysis in xenografts with and without carboplatin treatment on Day 2. Data for each spot represents the average value of each single sample with the number of nuclei analysed per sample ranging from 16 to 213 (average 103). Mann-Whitney U test (2-tailed); *P<0.05, **P<0.01 **D.** Changes in chromatin patterns in PEO1 cells after cisplatin (6uM) or radiation (6Gy) treatment for 24h, and in OV1002 xenografts in vivo after carboplatin treatment, measured by parameters describing nuclear texture using Image J. The (+) and (−) represent positive and negative correlations with each type of chromatin pattern, respectively, and the arrows indicate the direction of change for each pattern.

We next sought to establish whether similar nuclear changes occur *in vivo* using a platinum-sensitive OV1002 patient-derived ovarian cancer xenograft model [31]. Carboplatin, a cisplatin analogue, was used as this drug is commonly used clinically. After a single treatment with carboplatin, ovarian cancer xenografts were collected on days 0, 1, 2, 4, 7, and 14. Haematoxylin and eosin (H&E) staining and light microscopy indicated similar morphological changes to those seen *in vitro* (Figure 1). Untreated tumors tended to have strongly stained and homogeneous nuclei, while nuclei after carboplatin treatment had more lightly stained nuclei and greater heterogeneity (Figure 2B). When nuclear texture was analysed by image analysis, texture parameters were different in samples after carboplatin treatment compared to controls, with the most significant effects of single-dose carboplatin typically seen two days after treatment (entropy and IDM p=0.034 and 0.008, respectively; Figure 2C), indicating that chromatin pattern changes occur after platinum treatment *in vivo* and *in vitro* (Figure 2D).

### HDACs are differentially expressed in platinum-resistant cell lines

**Figure 3:**
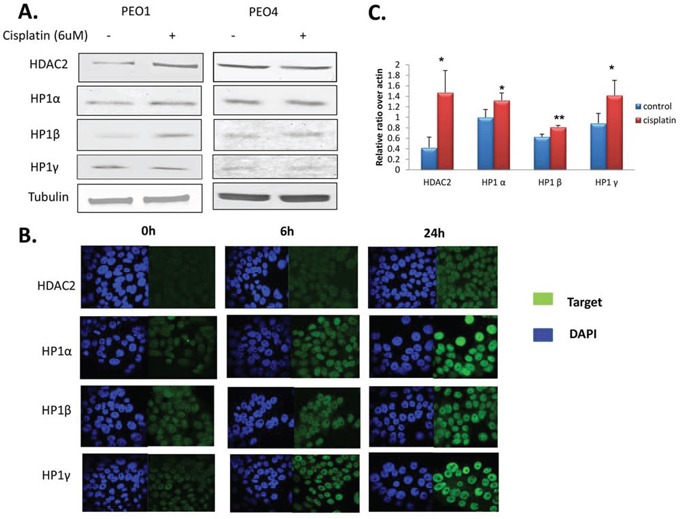
Expression of HDAC2 and heterochromatin proteins after cisplatin incubation **A.** Western blots for HDAC2, HP1α, HP1β, and HP1γ in PEO1 and PEO4 cells with or without cisplatin treatment (6 uM, 24h). Membranes were probed with the indicated antibodies, and tubulin was used as a loading control. **B.** HDAC2 and HP1 protein expression detected by immunofluorescence. Cells were seeded on cover slips and fixed as described in the Materials and Methods in PEO1 cells after cisplatin treatment for 6h and 24h. Alexa488 (green channel) and DAPI (blue channel) were used to stain target proteins and the nuclei, respectively. Images were taken using a confocal microscope. **C.** Expression of HDAC2, HP1α, HP1β, and HP1γ mRNA in PEO1 cells measured by RT-PCR as described in the Materials and Methods. Relative expression of the target gene was calculated as the average ΔCt and normalized to that of the housekeeping gene β-actin. Results are as presented as mean ±SD from biological triplicates. *p<0.05, **p<0.01 (Student's t-test).

It has previously been shown that HDAC1, HDAC3, and HDAC4 might be associated with resistance to chemotherapy and poor prognosis in cancer patients [32–34]. To investigate whether HDACs are involved in DNA damage-based treatment, we measured protein expression of HDAC class I (HDAC1, 2, 3, and 8) and IIA (HDAC4) members in PEO1 and PEO4 cells 24h after cisplatin treatment. The PE04 cell line was derived from the same patient as the PE01 cell line but after resistance had developed [30]. Since changes in nuclear texture after cisplatin treatment were most pronounced at 24 h, we speculated that HDACs would similarly show maximal changes at this time point. HDAC2 expression was increased approximately 1.5 fold in cisplatin-treated PEO1 cells, but not in PE04 cells, compared to controls at 24 h. Expression of HDACs 1, 3, 4 and 8 were unchanged in both cell lines after 24h (data not shown). This suggests that HDAC2 might be a cisplatin response biomarker *in vitro*, at least in sensitive cells. HP1 heterochromatin isoforms were also measured after cisplatin treatment (Figure 3A). Two HP1 isoforms (HP1α and HP1β) increased by about 30% and 70%, respectively, but only in PEO1 cells after 24 h cisplatin treatment, while HP1γ protein remained static.

To visualise and confirm these observations, immunofluorescence (IF) was performed on PEO1 and PEO4 cells with or without cisplatin treatment using antibodies targeting HDAC2, HP1α, HP1β, and HP1γ (Figure 3B). As expected, expression of HDAC2 increased 24 h after cisplatin treatment, and HP1 proteins gradually increased over 24 h of cisplatin treatment in PEO1 cells (Figure 3B). Again no changes were observed in PEO4 cells (data not shown). The IF images confirmed nuclear localisation of these targets.

Since HDAC2 and HP1 protein expression changed after cisplatin treatment in PEO1 cells, we next measured mRNA expression. Transcription of *HDAC2*, *HP1α*, *HP1β*, and *HP1γ* were all significantly elevated by cisplatin treatment (p<0.05) after 24 h in PEO1 cells (Figure 3C), mirroring the protein expression changes; however, mRNA levels remained unchanged in PEO4 cells (data not shown). The changes in HP1 expression suggest that the amount of heterochromatin increases after DNA-damaging treatment.

### Profiling expression of HDAC and HP1 proteins in ovarian cancer xenograft models

**Figure 4:**
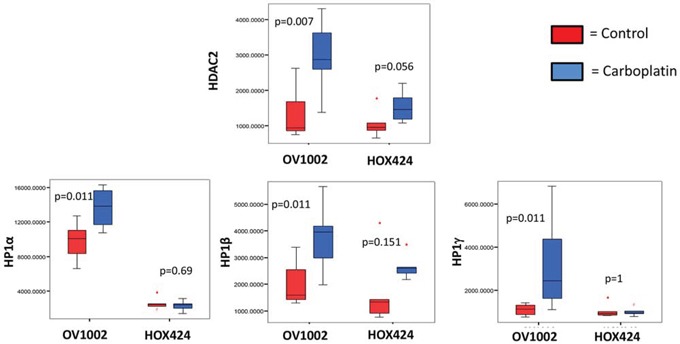
Expression of HDAC2, HP1α, HP1β and HP1γ in the OV1002 and HOX424 ovarian xenograft tumor models on Day 7 after carboplatin treatment The sample TMA was probed with the indicated antibodies and expression levels were quantified by AQUA analysis. Boxplots depict AQUA scores representing the expression of proteins. Data was compared between control group (red bar) and carboplatin-treated group (blue bar) within the platinum sensitive model (OV1002) and resistant model (HOX424), respectively. Man-Whitney analysis was performed and P values are indicated.

We then examined HDAC and HP1 expression in the platinum-sensitive OV1002 and platinum-resistant HOX424 xenograft models [31]. HDAC2 and all three HP1 proteins were significantly increased in the sensitive model (OV1002) after carboplatin treatment, with the most significant changes (p<0.05) observed on day 7 (Figure 4). In the HOX424 model, expression of these proteins was similar between control and treated groups.

### Time-dependent cellular DNA damage response induced by cisplatin in ovarian cancer cells

**Figure 5:**
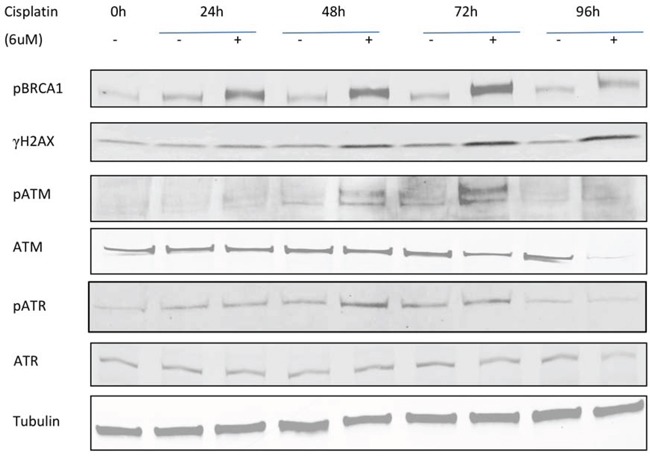
Time-dependent expression of DNA damage response proteins in PEO1 cells Cells were seeded and treated with or without cisplatin (6μM), and protein lysates were collected every 24h after treatment from 0h to 96h. Western blotting was performed to detect expression of pBRCA1, γH2AX, pATM, ATM, pATR, and ATR. Membranes were probed with the indicated antibodies, and tubulin was used as a loading control. Experiments were performed at least three times acquiring similar results. Blots from one representative experiment are shown.

We next investigated several DNA damage response (DDR) pathway members (γH2AX, pBRCA1, ATM, pATM, ATR, and pATR) by western blotting (Figure 5). As expected, the DNA damage response proteins pBRCA1, γH2AX, pATM, and pATR participated in the response to cisplatin and were upregulated after treatment. pBRCA1 expression increased after 24 h of cisplatin treatment in PEO1 cells, which persisted to 96 h, while γH2AX, pATM, and pATR increases occurred slightly later from 48 h. ATM and ATR protein expression remained stable except for ATM reductions at 96 h.

### Expression profiling of other HDAC family members, heterochromatin proteins, and DNA damage response proteins under HDAC2 suppression

**Figure 6:**
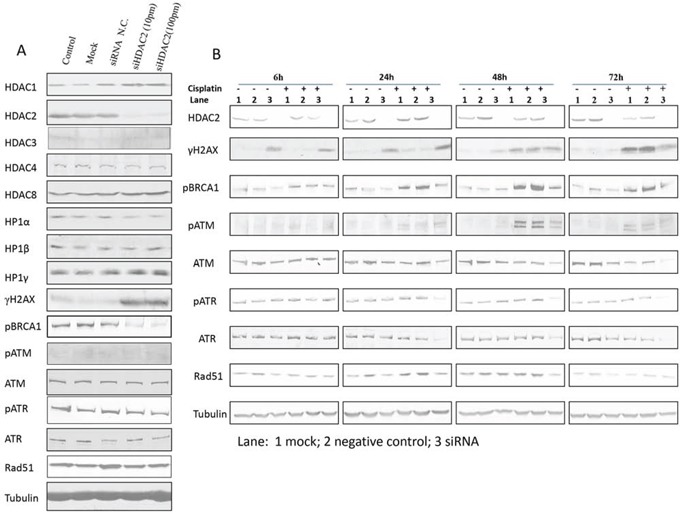
The effect of siHDAC2 knockdown on expression of other HDAC family members, HP1s, and DNA damage response proteins by western blotting in PEO1 cells alone (A) and in the presence / absence of cisplatin (B) Protein was lysed after HDAC2 was knocked down after 72 h using reverse transfection. Non-transfection (control), mock, and siRNA negative control were used as controls. Membranes were probed with the indicated antibodies, and tubulin was used as a loading control. Experiments were performed at least three times acquiring similar results. Blots from one representative experiment are shown. In (B), cells were treated with cisplatin (6μM) after 72h incubation with mock (lane 1), siRNA negative control (lane 2), or HDAC2 siRNA duplexes (10pmol in 6mL, lane 3). Protein lysates were collected at 6h, 24h, 48h, and 72h after cisplatin treatment and analysed by western blotting of DNA damage response proteins; tubulin was used as loading control. Experiments were performed at least three time acquiring similar results. Blots from one representative experiment are shown.

Given that HDAC2 expression showed the most pronounced changes in response to cisplatin, we examined the effect of HDAC2 knockdown by siRNA (Figure 6A). HDAC2 knockdown was efficient (Supplementary Figure 1). Expression of HDAC3, HDAC4, and HDAC8 were not significantly affected by HDAC2 knockdown. Interestingly, HDAC1 expression was mildly upregulated after HDAC2 knockdown, indicating a possible compensatory effect as previously reported [35–37]. Since HDAC2 was implicated in heterochromatin formation, we further assessed HP isoform expression after HDAC2 knockdown (Figure 6A). There were minor changes in HP1 protein expression, with mild downregulation (20%) of HP1α on HDAC2 knockdown. With respect to DNA damage response protein expression, HDAC2 knockdown resulted in marked upregulation of γH2AX and downregulation of pBRCA1 (Figure 6A). In contrast, other DDR proteins (pATM, ATM, pATR, ATR, and Rad51; Figure 6A) were not obviously affected. This suggests that double-strand breaks accumulate and DNA repair might be suppressed on HDAC2 knockdown, although the upregulation of γH2AX might also indicate that the cells are undergoing apoptosis [38].

### Characterisation of cellular responses to cisplatin treatment in ovarian cancer cells when HDAC2 is suppressed

We next investigated the potential role of HDAC2 in cisplatin response. PE01 cells were treated with cisplatin after HDAC2 knockdown (Figure 6B). HDAC2 was consistently upregulated at 24 h and downregulated at later time points in response to cisplatin without HDAC2 knockdown. Unsurprisingly, the induction of double-strand breaks (DSBs) indicated by γH2AX expression occurred as early as 6 h, while γH2AX expression increased after 24 h of cisplatin treatment in cells depleted of HDAC2 compared to cisplatin-treated or HDAC2 knockdown groups. This accumulation diminished over 72 h. As expected, pBRCA1, pATM, pATR, and Rad51 participated in the DNA damage response triggered by cisplatin at certain time points (6h, 24h, 48h, and 24h) and were upregulated. In contrast, cisplatin reduced expression of these proteins in cells with HDAC2 knockdown from 24h to 72h (Figure 6B).

The cisplatin-resistant PEO4 cell line was next studied to further clarify the involvement of HDAC2 during cisplatin-induced DNA damage responses (Supplementary Figure 2). There was little change in HDAC2 expression in PEO4 cells during cisplatin treatment, except for decreased expression at 72 h. In contrast to PEO1 cells, the cumulative effect of HDAC2 depletion and cisplatin treatment on γH2AX was not observed in PEO4 cells, although induction of DSBs was noted from 24 h in response to cisplatin alone. Additionally, pBRCA1, pATM, and RAD51 were upregulated by cisplatin treatment alone. Expression of these proteins was suppressed by HDAC2 depletion, although the pattern of expression over time was different to PEO1 cells.

### Role of HDAC2 in γH2AX foci formation during cisplatin treatment

**Figure 7:**
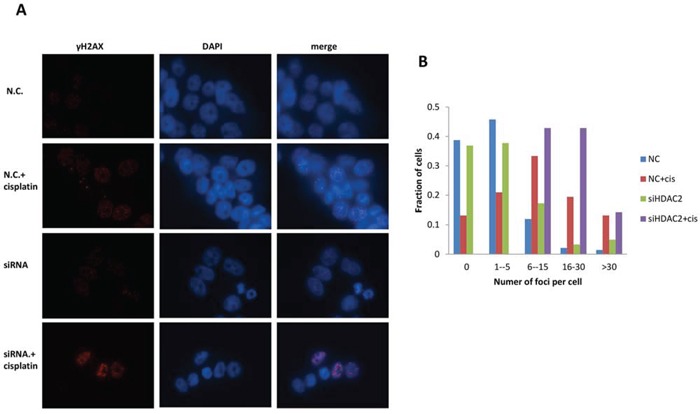
Immunofluorescence for γH2AX foci in PEO1 cells Cells were grown and treated as described before, and images were taken after 24 h treatment of cisplatin in cells with or without HDAC2 siRNA. Antibodies against H2AX phosphorylation at Ser 139 were used to probe cellular γH2AX foci (red channel), and DAPI was applied for nuclear staining (blue channel). **A.** Representative images from one experiment are shown. >100 cells in each group were included for one experiment, and three independent experiments were performed. **B.** Distribution of γH2AX foci / cell in PEO1 cells.

γH2AX foci at DNA damaged domains are indicative of inter-strand crosslinking by cisplatin [39]. The detection of ser139-phosphorylated γH2AX foci has been widely used to evaluate double-strand breaks (DSBs) [40]. Therefore, γH2AX foci were targeted and visualised using immunofluorescence microscopy (Figure 7). γH2AX foci were formed in PEO1 (Figure 7) and PE04 cells in response to cisplatin (Supplementary Figure 3). Similarly, HDAC2 knockdown resulted in γH2AX foci in both cell lines (Figure 7 and Supplementary Figure 3). There was a marked increase (number and intensity) in γH2AX foci in PEO1 cells treated with cisplatin after HDAC2 knockdown, which was less pronounced in PEO4 cells, similar to cisplatin alone (Supplementary Figure 3). Using a cut-point of five foci per cell as negative background, the number of foci-positive PEO1 cells significantly increased after cisplatin treatment, HDAC2 knockdown, and cisplatin treatment in HDAC2-depleted cells (p<0.05 and 0.01 compared to cisplatin-treated and siRNA-transfected groups, respectively; Figure 7). In PE04 cells, cisplatin induced foci (p<0.001), while HDAC2 knockdown did not.

### Cell fate determination by HDAC2 knockdown during cisplatin treatment

**Figure 8:**
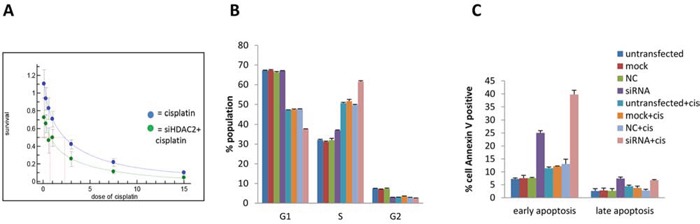
The effect of HDAC2 knockdown on cell number (A), cell cycle distribution (B), and apoptosis (C) in PEO1 cells A SRB assay profile for growth inhibition of cisplatin on PEO1 cells with (green line) and without (blue line) HDAC2 knockdown. Cells were reverse transfected with HDAC2 siRNA, followed by treatment with cisplatin for 72h. Three controls were included as described before. B. Percentages of the populations selected in G0/1, S, G2/M phases of the cell cycle were detected by flow cytometry. Columns represent the mean percentage of triplicate independent samples. Error bars represent SD. The Brown-Forsythe test followed by Games-Howell post-hoc test were performed to compare groups for each phase. C. The effect of HDAC2 knockdown on apoptosis in PEO1 cells using the annexin V assay is shown. Cell number percentages of the population selected with positive annexin V staining were detected by flow cytometry after cisplatin treatment for 72h, and data are separated into early and late apoptosis based on propidium Iodide (PI) signal. Columns represent the mean percentage of triplicate independent samples. Error bars represent SD. One-way ANOVA analysis was performed to compare data among groups, and the Tukey HSD pot HOC test was used to compare groups.

We assessed the effect of HDAC2 knockdown on cell fate with and without cisplatin treatment. Growth inhibition by cisplatin was concentration dependent manner in both control HDAC2 knockdown groups in PEO1 cells (Figure 8A). Cisplatin treatment alone induced significant S-phase arrest in PEO1 cells (+160%, p<0.001) and decreased the number of G1-phase cells (−30%; p<0.05) (Figure 8B). HDAC2 knockdown alone increased the S phase population (+120%, p<0.05). As expected, cisplatin treatment in HDAC2-depleted cells altered the cell cycle distribution in PEO1 cells, but in a different way: HDAC2 knockdown caused further S-phase arrest induced by cisplatin in PEO1 cell (+120%, p<0.05) and an additional reduction in G1 phase cells (−20%, p<0.001). In contrast, HDAC2 depletion seemed to reduce S-phase accumulation (−20%, p<0.001) and increase the G1-phase population (+140%, p<0.05) based on measurements of cisplatin treated cells without transfection.

We next analysed the effect of cisplatin and HDAC2 knockdown on apoptosis (Figure 8C). Cisplatin induced early apoptosis (annexin V positive only) (p<0.001). HDAC2 knockdown caused both early and late apoptosis (p<0.001). Furthermore, HDAC2 depletion induced significant (especially early) apoptosis after cisplatin treatment (p<0.001).

### Reversibility of HDAC inhibition on nuclear morphological changes during cisplatin treatment

**Figure 9:**
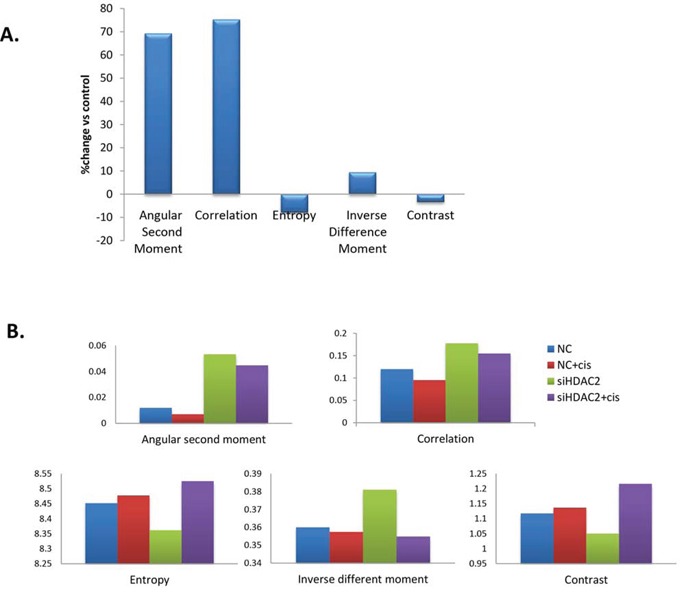
Changes in nuclear texture features in PEO1 cells treated with TSA (A) or transfected with HDAC2 siRNA before cisplatin treatment (B) A. PEO1 cells were grown on coverslips and treated with TSA (250 nM) for 24h, and nuclei were stained with DAPI for visualisation using a fluorescence microscope. At least 100 nuclei were included in one experiment. Nuclear texture was analysed by measuring five texture parameters (angular second moment, correlation, entropy, inverse different moment, and contrast) in Image J software. Data are presented as the average change (%) in TSA treated group for each parameter over control group. B. PEO1 cells were grown on coverslips, transfected with HDAC2 siRNA for 72h, and treated with cisplatin (6μM) for a further 24h. Nuclei were stained with DAPI for visualisation using fluorescence microscopy. At least 100 nuclei were included in each group in one experiment. Nuclear texture was analysed by measuring five texture parameters (angular second moment, correlation, entropy, inverse different moment, and contrast) using Image J.

To test if the nuclear structural changes were mediated by HDACs, then specifically HDAC2, we investigated whether the nuclear changes were reversible by applying either the broad-spectrum HDAC inhibitor trichostatin A (TSA) or HDAC2 siRNA in PEO1 cells. When PEO1 cells were treated with TSA, all five texture parameters were altered: angular second moment, correlation, and inverse difference moment increased, while entropy and contrast decreased (Figure 9A). Decreased chromatin heterogeneity and contrast and increased homogeneity are consistent with HDAC inhibition relaxing the chromatin structure. With HDAC2 siRNA (Figure 9B), comparable changes to TSA were observed.

## DISCUSSION

Within this study, we show that comparable nuclear morphological changes occur in several ovarian tumor models (cell lines and xenografts) after treatment-induced cellular damage. Image analysis of nuclear texture has previously been used to differentiate between benign and malignant cancers [28], examine apoptotic cells [41], and study the condensation and distribution of chromatin in the nucleus in drug-sensitive and resistant cells [42]. In the present study, increased nuclear heterogeneity was assumed to indicate a transition from euchromatin to heterochromatin. Consistent with this, HP1 isoform expression increased after cisplatin treatment *in vitro*, which was accompanied by enhanced expression of HDAC2 at both the mRNA and protein levels. Studies *in vivo* using a patient-derived ovarian cancer xenograft model also showed elevated HDAC2 and HP1 isoform expression after chemotherapy.

As an essential component of heterochromatin, HP1 accumulates in response to UV or ionising radiation consistent with chromatin reorganisation [43]. We obtained a similar result, with enhanced heterochromatin formation following chemotherapy both *in vivo* and *in vitro*. HDAC2 was upregulated after 24h of cisplatin treatment *in vitro*, with similar results seen *in vivo* in ovarian cancer xenografts. In line with its function as a regulator of condensed chromatin formation, HDAC2 expression changed consistent with the observed changes in nuclear texture and was a possible mediator of the DNA damage response. This alteration in chromatin pattern might also indicate that chemotherapy induces transcriptional silencing, perhaps as a form of cellular self-protection upon injury. There is evidence that HDAC-containing complexes including Mi-2/NuRD and/or Sin3/HDAC chromatin-modifying complex (containing HDAC1 and HDAC2) participate in nuclear reorganisation and gene repression during development [44]. Mi-2/NuRD and mSin3/HDAC co-repressor complexes are also necessary for pericentric heterochromatin assembly and chromosome segregation [45, 46].

There were time-dependent changes in the expression of DNA damage response proteins including pBRCA1, γH2AX, pATM, and pATR after cisplatin treatment in PEO1 cells. As a marker of double strand breaks, elevated γH2AX expression indicated activation of DSB repair pathways in response to cisplatin treatment in PEO1 cells; there is evidence to show that the tumor suppressor function of BRCA1 occurs via heterochromatin silencing, with increased levels of both heterochromatin and BRCA1 observed after DNA damage [47]. Our results are consistent with these findings, and suggest that chromatin remodelling by HDACs is involved in the cellular response to DNA damage therapy such as double strand break and DNA repair.

HDACs are associated with malignancy and poor clinical outcomes in multiple cancer types [13, 17]. Roles for HDAC2 have been identified in a variety of malignancies. In colon cancer cells, HDAC2 expression has been associated with chemoresistance to genotoxic stress [48]. In neuroblastoma, HDAC2 has been show to act with N-MYC to reduce TP53INP1 expression which influences p53 phosphorylation at serine 46, with subsequent effects on cell proliferation and survival [49]. In leukemia, HDAC2 silencing induces modulation of gene expression leading to strong transcriptional activation [50] while in lung cancer, HDAC2 has been proposed to exert an effect on survival by sustaining Mdm2-survivin levels [51]. A number of studies have demonstrated efficacy of combined HDAC inhibitor and cytotoxic chemotherapy. The HDAC inhibitor romidepsin (FK228), approved for phase I and II trials, enhances the cytotoxic effects of cisplatin by reducing cell growth and inducing more DNA damage-induced cell death *in vitro* and *in vivo* [52]. Since HDAC2 in particular was associated with early responses to cisplatin in our cell culture studies, cell growth was evaluated in response to HDAC2 knockdown. HDAC2 depletion reduced the IC_50_ of cisplatin in PEO1 cells, suggesting that HDAC2 loss enhances the effect of cisplatin treatment. Repression of cell growth in chemo-sensitive PEO1 cells by cisplatin after HDAC2 silencing appeared to be due to accumulated S-phase arrest and apoptosis.

Cell cycle progression blockade has been reported to occur as a result of checkpoint activation during DNA damage-based therapy, and at least one checkpoint protein, Chk1, is upregulated during intra-S-phase accumulation by affecting chromatin formation and interfering with the initiation and elongation of DNA replication [53]. It is feasible that HDAC2 interacts with checkpoint proteins to prevent DNA replication by condensing the chromatin in self-protection, and triggers apoptosis via distinct pathways in platinum sensitive and resistant cells. Several mechanisms have been suggested to explain HDAC inhibitor-induced apoptosis, such as altered transcription and DDR and DNA damage repair [54–56]. The early upregulation (after 24 h) of HDAC2 in sensitive cells may suggest that HDAC2 is acting as a sensor of DNA damage and a trigger of downstream DDR events (such as activation of ATM, ATR, and BRCA1) and chromatin remodelling, followed by histone hyperacetylation to relax the chromatin structure and facilitate recruitment of more DDR mediators to the damaged site.

DSBs (measured by γH2AX) accumulated after 24 h of cisplatin treatment in HDAC2-depleted PEO1 cells, but not PEO4 cells. High γH2AX expression has previously been associated with cell viability and apoptosis in ovarian cancer [57], which was attributed to the cell type tested and time after damage. H2AX activation has also been noted a number of days after treatment with an HDAC inhibitor, suggesting that increased γH2AX expression precedes cancer cell death [58]. Suppression of DDR activation (as evidenced by pATM, pATR, pBRCA1, and RAD51 expression) after HDAC2 knockdown strongly suggests that HDAC2 is involved in responses to DNA damage-based treatment. The differences between PEO1 and PEO4 cells in DSB accumulation and time of pathway activation or suppression is consistent with the observed differences in cell cycle progression, and also might be due their differences in cisplatin sensitivity. The two major pathways of DSB repair, non-homologous end joining (NHEJ) and homologous recombination (HR), appear to compensate for each other [59], and their balance might be disrupted by the known BRCA2 deficiency in PEO1 and secondary mutation to restore BRCA2 in PE04 [60]. This might influence the dominant mechanism of repair in the two cell lines and cause differences in the observed functional activity of the measured DNA damage response proteins after cisplatin treatment.

Together, these results suggest that chromatin remodelling caused by increased HDAC2 expression might be an early cellular event (within 24 h) in response to DNA damage. We postulate that, in sensitive tumors, early alterations in chromatin induced by chemotherapy and mediated by histone deacetylation are a form of cellular self-defence to injury by repressing transcription, initiating chemotherapy-triggered DDR, and promoting survival. This is followed by a change to a relaxed chromatin conformation by histone hyperacetylation, such as via H3k56Ac and H4k16Ac, to provide accessibility of DNA to downstream proteins at damaged sites [61–63]. However, resistant tumors behave differently in terms of their response to DNA damage to chemotherapy; this might be due to their initial chromatin environment or due to other changes in the components of the DDR response pathways in which HDACs participate. In one study, HDAC1/2-associated immediate histone hypoacetylation occurred after laser microirradiation of a human osteosarcoma cell line to promote NHEJ, which was followed by hypoacetylation to enhance HR and guard genome integrity [64], supporting our hypothesis. Linkage between HDAC2 modulation and H4k16 acetylation has been shown in breast cancer [65].

By identifying the detailed roles that HDACs play in DNA damage responses by remodelling chromatin, we hope to better understand the molecular processes that underpin nuclear structure and identify novel mechanisms that control responses to chemotherapy.

## MATERIALS AND METHODS

### Cell culture

Cisplatin-sensitive PE01 and cisplatin-resistant PE04 ovarian cancer cell lines derived in our laboratory [30] were cultured as monolayers in RPMI 1640 supplemented with 10% heat-inactivated foetal calf serum (FCS) and penicillin/streptomycin (100 IU/mL) in 5% CO_2_ at 37°C.

### Ovarian cancer xenografts

Two ovarian cancer patient-derived xenograft models were previously established in our laboratory: OV1002 and HOX424 [66]. Female adult CD-1 nude mice housed in individually ventilated cages were treated with carboplatin (50 mg/kg i.p.) on day 0, and tumor samples were collected on days 0, 1, 4, 7, and 14 after treatment. Tumors were formalin fixed and paraffin embedded (FFPE). The OV1002 xenograft model was markedly more sensitive to carboplatin treatment than the HOX424 model [31]. Xenograft studies were undertaken under a UK Home Office Project Licence in accordance with the Animals (Scientific Procedures) Act 1986, and the University of Edinburgh Animal Ethics Committee approved the study protocol.

### Nuclear texture analysis

Confocal microscopy images were obtained using a Nikon A1R Confocal Microscope (Nikon Corporation, Tokyo, Japan) and images were viewed in NIS Viewer (Nikon). Cells were grown on chamber slides for the periods specified and fixed in 10% formalin in PBS for 10 min. After washing three times with PBS-0.05% Tween 20, slides were counterstained with anti-fade reagent with DAPI (Invitrogen, P36931; Thermo Fisher Scientific, Waltham, MA). Nuclear texture was analysed using Image J software. Briefly, after input of 8-bit images, fully focused areas containing tumor cells were marked as regions of interest (ROI) and individual nuclei selected. Nucleus counter and GLCM (grey-level co-occurrence matrix) [67] manager plugins were performed on each image and five parameters associated with texture obtained: correlation, contrast, angular second moment (ASM), inverse different moment (IDM), and entropy. GLCM [67, 68] is a second-order texture calculation that considers the distance and angle relationship between two-pixel groups in the original greyscale image under the same intensity of grey pixels within a defined area. Texture features were regarded as positively or negatively associated with chromatin patterns (homogeneity, heterogeneity, and contrast) as previously described [28, 29].

### siRNA knockdown of HDAC2

Loss of HDAC2 function was achieved with siRNA transfection of cells in 60mm cell culture dishes according to the manufacturer's instructions. Briefly, siRNA duplex-Lipofectamine^™^ RNAiMAX complexes (Life Technologies, Thermo Fisher Scientific) were prepared as follows. 10-100 pmol siRNA duplex was diluted in 500μl Opti-MEM^®^ I Medium (Life Technologies) without serum in each cell culture dish and mixed gently. The duplex sequences were: GACAAACCAGAACACUCCAGAAUAU and AUAUUCUGGAGUGUUCUGGUUUGUC. A negative scrambled stealth siRNA duplex with similar GC content (low GC duplex) to the target was used as control. 0.8 μl Lipofectamine^™^ RNAiMAX was added to each dish containing diluted siRNAs and incubated at room temperature for 20 min. After incubation, cells were diluted in complete growth medium without antibiotics at 700,000 – 800,000 cells/5mL to ensure a cell density of 30-50% 24h after seeding, and 5 mL was added to each well. Controls were untransfected (no transfection agents), mock (only Lipofectamine^™^ RNAiMAX mixture), and negative control (random RNAi duplex). The cells and the complexes were incubated for 24-120 h at 37°C in full serum without antibiotics. For drug treatment, cisplatin was added after 48 h of transfection and cells were collected after several time points as indicated.

### Protein extraction from mammalian cell lines

Cultured cells were washed in cold PBS and lysed by scraping in ice-cold isotonic lysis buffer (50mM Tris-HCl (pH7.5), 5mM EGTA (pH 8.5), 150mM NaCl, 1% Triton X-100) supplemented with aprotinin (10 μg/mL) and a cOmpleteTM Protease Inhibitor Cocktail Tablet (Roche, 11836153001) for 30 min on ice. Lysates were centrifuged for 6 min at 13,000 × g and the supernatant stored at −70°C. Protein concentrations were determined using the bicinchoninic acid (BCA) assay (Sigma, BCA-1; Sigma-Aldrich, St. Louis, MO).

### Western blotting

After SDS-PAGE using 10% polyacrylamide gels, resolved proteins were transferred to nitrocellulose membranes at 30V, 4°C overnight. After transfer, membranes were rinsed in PBST and blocked with Li-Cor Odyssey Blocking Buffer (LI-COR Biosciences, Lincoln, NE; diluted 50:50 in PBS) for 1h at room temperature before probing overnight at 4°C with the appropriate primary antibody in Li-Cor Odyssey Blocking Buffer. Primary antibodies were rabbit and obtained from Cell Signaling Technology (Beverly, MA) and used at 1:1000 unless otherwise indicated: anti-HP1 alpha (#2623), anti-HP1 beta (Abcam/ab10478), anti-HP1 gamma (#2619), anti-HDAC1 (mouse, #5356), anti-HDAC2 (#2540), anti-HDAC3 (Abcam (Cambridge, UK) ab32369), anti-HDAC4 (Abcam ab32534), anti-HDAC8 (Abcam ab39664), anti-AMT (1:750 Abcam ab67998, mouse), anti-pATM (Ser1981; #4526), anti-ATR (Abcam ab2905), anti-pATR (Ser428; #2853), anti-pBRCA1(Ser1524; #9009), anti-γH2AX (#2577), anti-Rad51 (H-92) (Santa Cruz Biotechnology, Dallas, TX; sc-8349), anti-α-tubulin (1:6000 Mouse Abcam ab7291), anti-β-tubulin (1:6000 Abcam ab6046), anti-GAPDH (1:8000 Mouse Abcam ab8245). Membranes were washed with PBS-Tween20 before incubation with fluorescently-labelled secondary antibodies diluted in Odyssey Blocking Buffer (50:50 in PBST) at 1:10,000 dilution. Mouse-derived primary antibodies were detected using an anti-mouse fluorescently-labelled secondary antibody (680 nm wavelength), whilst rabbit-derived primary antibodies were detected using an anti-rabbit fluorescently-labelled secondary antibody (800 nm wavelength) (45 min incubation). By combining a mouse primary with a rabbit primary along with their respective secondary antibodies, dual-labelled blots were obtained. Membranes were scanned on the Li-Cor Odyssey scanner, and the fluorescence value (integrated intensity, I.I.) corresponded to protein expression levels. Alpha-tubulin (Mouse Abcam ab7291) was used as loading control.

### RNA preparation and real time polymerase chain reaction (RT-PCR)

Total RNA was extracted from cultured cells using the Qiagen Mini RNeasy Kit according to the manufacturer's instructions (Qiagen, Limburg, NL). The concentration and quality of RNA were assessed by NanoDrop. 1μg of total RNA from each individual sample was reverse transcribed using the QuantiTect Reverse Transcription kit (Qiagen) following the manufacturer's instructions to produce 20μL of cDNA, which was quantified using Rotorgene (Corbett Research, San Francisco, CA) and the QuantiTect SYBR Green system (Qiagen) following the manufacturers' instructions. For PCR, a 13-fold dilution of the cDNA mixture (10-fold dilution for standard curve) and a 10-fold dilution of primers for HDAC8 and β-actin (Qiagen) were used. A 15ul mixture of 7.5μl 2xQuantiTect SYBR Green iMaster Mix, 1.5 μL primer mix (0.3 μM), 2.5mM of MgCl_2_, and 1.5 μL cDNA was prepared in RNase-free water for the PCR reactions. PCR was performed as follows: 95°C for 15min; 45 cycles at 94°C for 15s, 56°C for 30s, 72°C for 30s; 72°C for 5min followed by melting from 55°C to 95°C at 0.2°C/s.

### Immunofluorescence (IF) on xenografts

4μm TMA sections were deparaffinised in xylene for 5 min and rehydrated through graded ethanol. For antigen retrieval, sections were treated with 0.15mM sodium citrate, pH 6.0 or Tris-EDTA, pH9.0 using a microwave pressure cooker for 5 min. Sections were rinsed in 0.05% PBST, blocked in 3% hydrogen peroxide and serum-free protein block (Dako, Glostrup, Denmark; #X0909), 10 min each. After blocking, slides were incubated with primary antibodies diluted in second primary antibody (mouse anti-cytokeratin, Invitrogen, #18-0132) in Dako antibody diluents (1:25) for 1h at room temperature or overnight at 4°C. Primary antibodies were rabbit from Cell Signaling unless otherwise indicated: anti-HP1 alpha (#2623; 1:100 dilution), anti-HP1 beta (Abcam ab10478; 1:150), anti-HP1 gamma (#2619; 1:400 dilution), anti-HDAC1 (mouse #5356; 1:25 dilution), anti-HDAC2 (#2540; 1/100 dilution), anti-HDAC3 (Abcam ab32369; 1/100 dilution), anti-HDAC4 (Abcam/ab32534; 1/100 dilution) and anti-HDAC8 (Abcam/ab39664; 1/100 dilution). Sections were rinsed in 0.05% PBST three times followed by incubation with secondary antibodies for 1 h at room temperature with a 1:25 dilution of goat-anti-mouse Alexa 555 antibody (Invitrogen, #A21422). After rinsing, sections were incubated with target signal amplification diluents and Cy5 tyramide at 1:50 in the dark for 10 min at room temperature for target visualisation. Finally, slides were rinsed, dehydrated in 80% ethanol for 1 min, air dried in the dark, and counterstained and cover-slipped using Prolong Gold anti-fade reagent with DAPI (Invitrogen, P36931).

IF was analysed using the Automated QUantitative Analysis (AQUA) system (HistoRx, New Haven, CT) as previously described [69]. For each immunofluorescence image, AQUAnalysis software evaluated the quantity (in AQUA units=Au) of target protein expression (through Cy-5-tyramide) within the cytoplasm (identified by cytokeratin) and nuclei (DAPI). Images were examined to exclude imaging faults and normal tissue, thus target protein expression was scored only in invasive cancers. Cores containing <5% epithelium were automatically excluded to ensure tissues were representative of tumors [70]. The final normalised AQUA score detecting the fluorescence correlates with the expression level of target protein.

### Sulforhodamine B cell proliferation assay

Cells were harvested in log phase, counted using a haemocytometer, and optimal initial numbers of cells in 200 uL per well were seeded into 96 well cell culture plates for 72h. After 0 to 6 days incubation with small molecules, cells were fixed in 25% cold trichloroacetic acid (50uL/well), and incubated for 1 h at 4°C. Plates were washed, air-dried, and stained with sulforhodamine B dye (0.4% solution in 1% acetic acid, 50 μl/well) for 30 min. After washing with 1% acetic acid, plates were dried. 100uL Tris buffer (10mM, pH 10.5) was added into each well 1 h prior to reading and the optical density (OD) was recorded using a Biohit BP800 Microplate reader (Biohit, Helsinki, Finland) at 540 nm.

### Cell cycle analysis

Cells were harvested and plated as described for western blotting. At the time points indicated, cells were trypsinised and transferred to 5 mL BD Falcon tubes (BD Biosciences). Citrate buffer (trisodium citrate (301287F, BDH Laboratory Supplies, Poole, UK), 121 mg Tris Base (T1378, Sigma), 1044 mg spermine tetrahydrochloride (S2876, Sigma) and 2mL Nonidet NP40 (N3516, Sigma) in 2000mL distilled water, pH7.6) was added after centrifugation. The following solutions were added in sequence prior to analysis: 450uL solution A (0.003% trypsin type IX-S (T0303, Sigma) in citrate buffer, pH7.6) for 2 min, solution B (0.05% trypsin inhibitor (T9253, Sigma) and 0.01% RNAse A (R4875, Sigma) in citrate buffer pH7.6) for 10 min, and solution C (0.0416% propidium iodide (81845, Sigma) and 0.1% spermine tetrahydrochloride (S2876, Sigma) in 500 mL citrate buffer pH7.6) for 10 min in the dark. Apoptosis was detected at 24 h using the TACS Annexin V-FITC Kits (R &D Systems) according to the manufacturer's protocol. Flow cytometry was performed using a BD FACSAriaII SORP (Becton Dickinson, Franklin Lakes, NJ). BD FACSDiva software (Becton Dickinson, Version 6.1.2) was used for instrument control and Flowjo software (Version 7.6.5) for Data analysis.

### Quantitative analysis of γH2AX foci formation in cells

Cells were grown and stained as described above using anti-phospho-histone H2AX (ser139, γH2AX; Millipore 05-636) primary antibody. After mounting with DAPI and air-drying, cells were visualised with a BriteMAC or MacRd microscope. Nuclear images were taken with IPLab software with single filters for each channel and the same exposure for each set of experiments. For each slide, over 100 nuclei were counted for foci formation. The number of γH2AX foci in each nucleus was counted by the PZ Foci EZ plugin in ImageJ as described (available at www.pzfociez.com). Briefly, a nuclear mask defining ROIs was first created for each channel, and then the foci number was automatically counted in the channel within the defined ROI.

### Statistical analysis

Student's t-test was used to compare two independent samples. One-way ANOVA followed by the Tukey test was for multiple comparisons of groups with equal variance. Pearson correlations were performed for IF correlations. The Wilcoxon signed-rank test was used to compare target protein expression differences between the pre- and post-treatment samples from patients, and the Mann-Whitney test was performed to compare xenograft data. All data were analysed using GraphPad Prism (GraphPad Software Inc., La Jolla, CA), and a p-value <0.05 was considered statistically significant.

## SUPPLEMENTARY FIGURES


