# Supplementary material for: The role of HDAC2 in chromatin remodelling and response to chemotherapy in ovarian cancer
Source: Oncotarget. 2015 Dec 14;7(4):4695–711. doi: 10.18632/oncotarget.6618 (PMC4826236; doi:10.18632/oncotarget.6618)
Supplement: Supplementary file 1 [file oncotarget-07-4695-s001.pdf]

SUPPLEMENTARY FIGURES

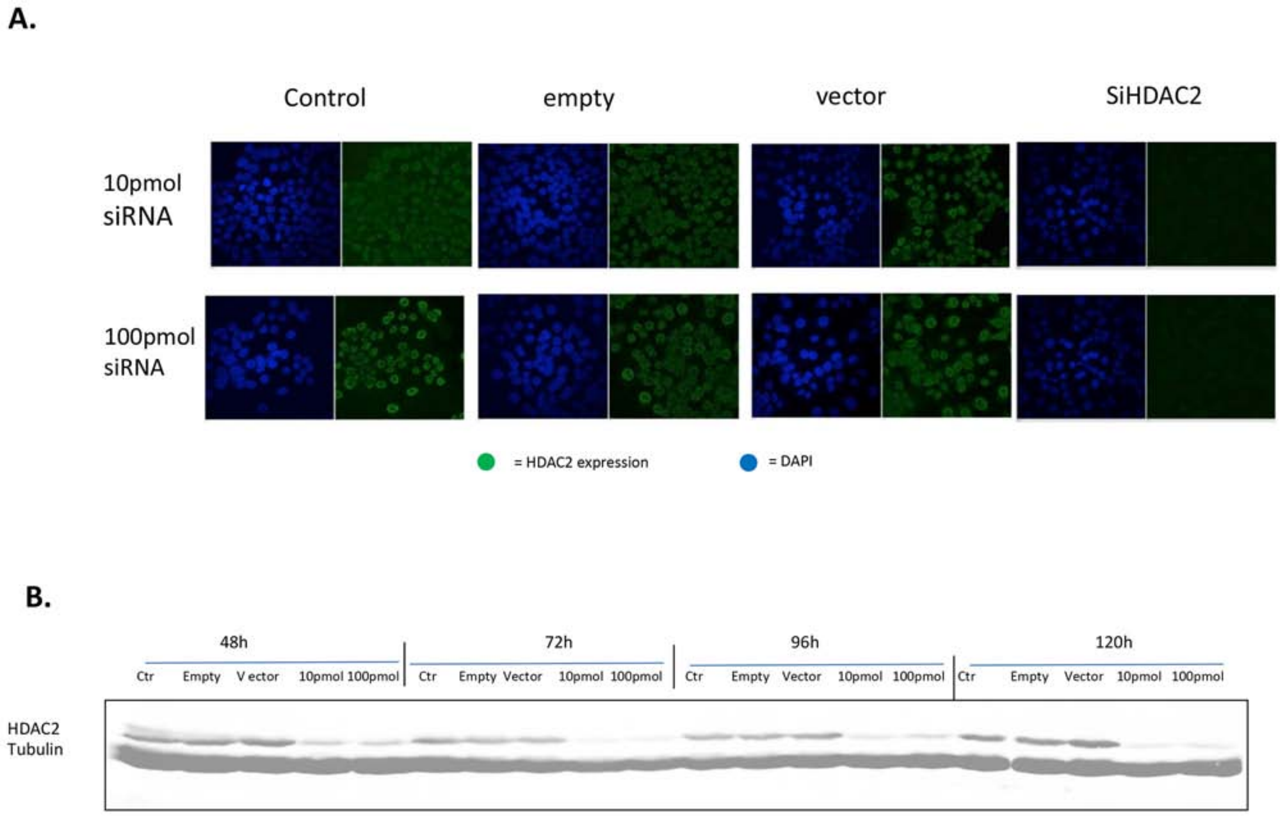

Supplementary Figure S1: Assessment of HDAC2 protein expression by immunofluorescence (A) or western blotting (B) after HDAC2-targeted siRNA transfection in PEO1 cells. A. Representative images for HDAC2 expression detected by immunofluorescence. PEO1 cells were seeded on cover glasses and fixed as described in Materials and Methods after siRNA transfection (10pmol or 100pmol in 6mL) for 72h, and untransfected, mock, and negative treatment were used as control groups. Alexa488 (green channel) and DAPI (blue channel) were used to stain target proteins and nuclei, respectively. Pictures were taken using a confocal microscope. B. Assessment of HDAC2 protein expression by western blotting after HDAC2-targeted siRNA transfection in PEO1 cells. Cells were reverse transfected (as described in the methods) with siRNA (10pmol or 100pmol in 6mL) targeting HDAC2, and untransfected group (Ctr), mock group, and negative control were included as controls. Total protein was extracted from the cells at 48h, 72h, 96h, and 120h after transfection. Western blotting was performed to detect expression of HDAC2. Membranes were probed with the indicated antibodies, and tubulin was used as loading control.

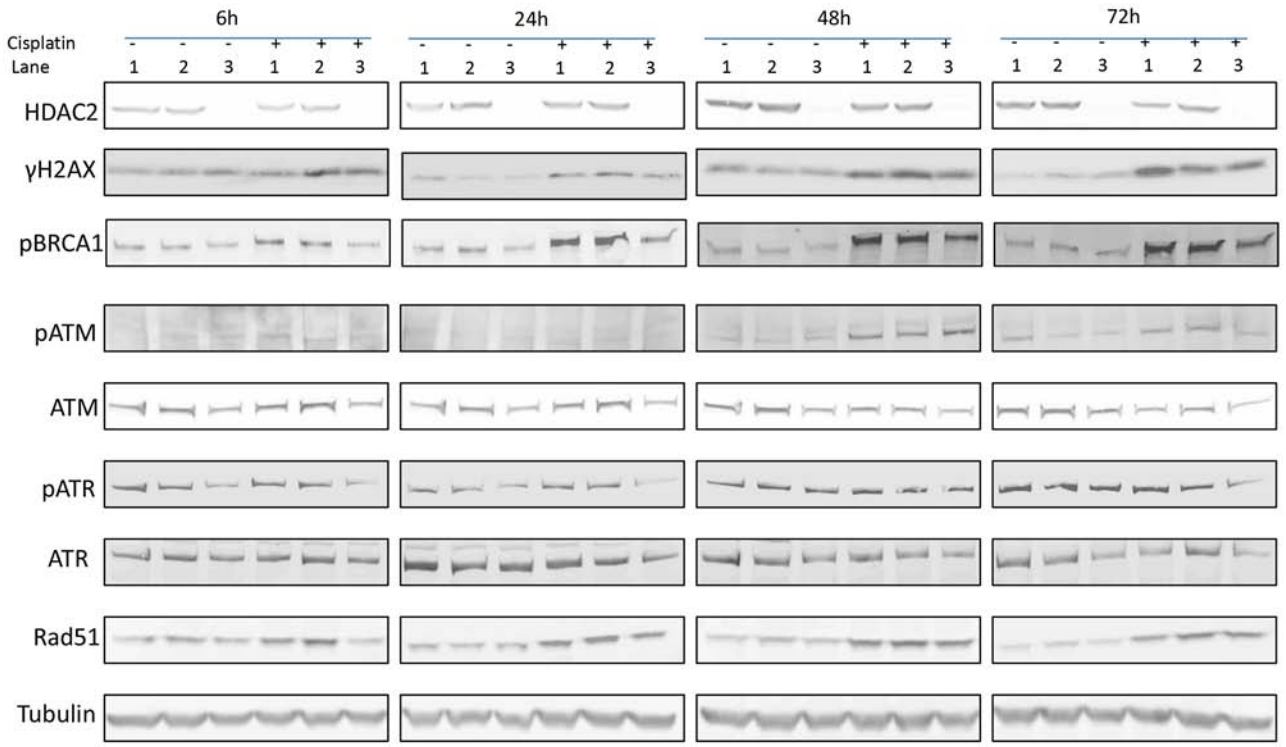

Supplementary Figure S2: The effect of siHDAC2 knockdown on expression of other HDAC family members, HP1s and DNA damage response proteins by western blotting in PEO4 cells in the presence / absence of cisplatin. Protein was lysed after HDAC2 was knocked down after 72h using reverse transfection. Non-transfection (control), mock, and siRNA negative control were used as controls. Membranes were probed with the indicated antibodies, and tubulin was used as a loading control. Experiments were performed at least three times, acquiring similar results. Blots from one representative experiment are shown. In (B), cells were treated with cisplatin (6μM) after 72h incubation with mock (lane 1), siRNA negative control (lane 2), or HDAC2 siRNA duplexes (10pmol in 6mL, lane 3). Protein lysates were collected at 6h, 24h, 48h, and 72h after cisplatin treatment and analysed by western blotting of DNA damage response proteins; tubulin was used as loading control. Experiments were performed at least three time acquiring similar results. Blots from one representative experiment are shown.

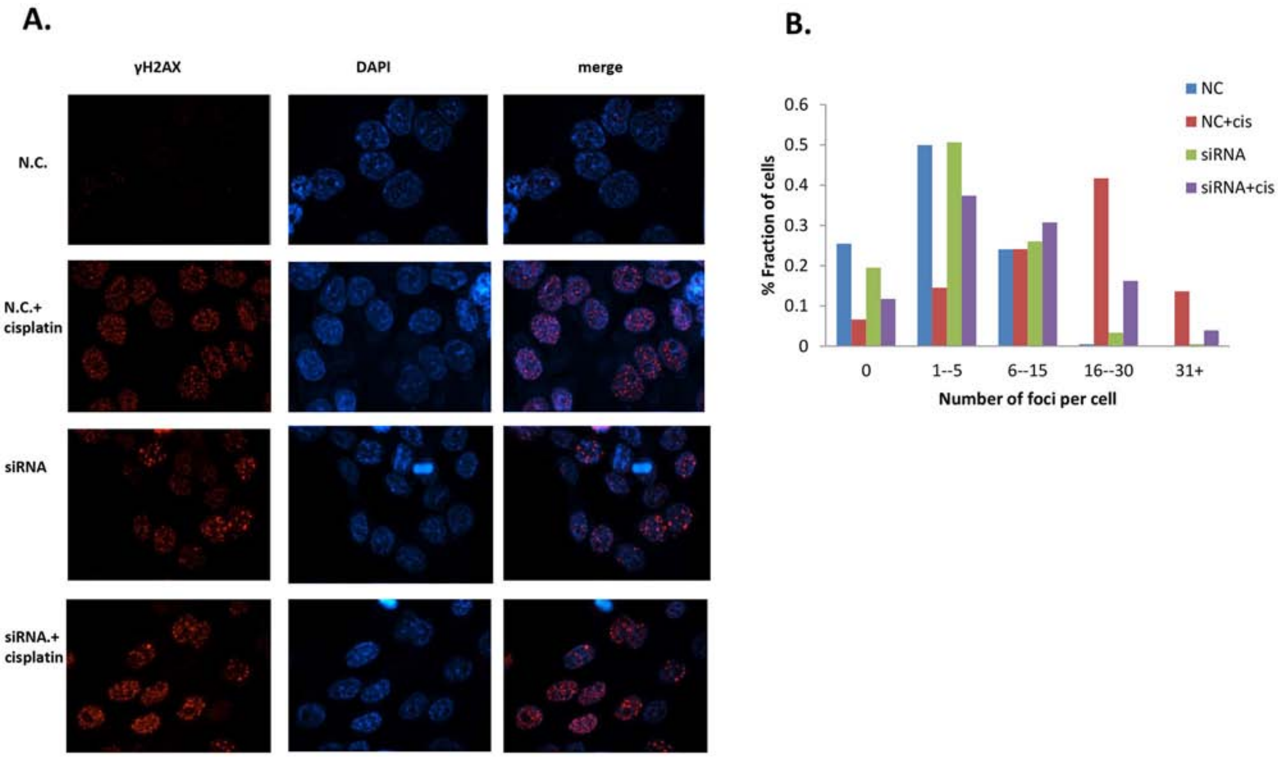

Supplementary Figure S3: Immunofluorescence for γH2AX foci in PEO4 cells. Cells were grown and treated as described before, and images were taken after 24h treatment of cisplatin in cells with or without HDAC2 siRNA. Antibodies against H2AX phosphorylation at Ser 139 were used to probe cellular γH2AX foci (red channel), and DAPI was applied for nuclear staining (blue channel). A. Representative images from one experiment are shown. > 100 cells in each group were included for one experiment, and three independent experiments were performed. B. Distribution of γH2AX foci / cell in PEO4 cells
